# Manual annotation based sentiment analysis of user feedback in health and wellness app reviews

**DOI:** 10.1038/s41598-025-28799-5

**Published:** 2025-12-29

**Authors:** Linda Varghese, Rajesh R. Pai, G. Savitha, S. Girisha, Naganna Chetty

**Affiliations:** 1 Manipal Institute of Technology, Manipal Academy of Higher Education, Manipal, India; 2 Department of Humanities and Management, Manipal Institute of Technology, Manipal Academy of Higher Education, Manipal, India; 3 Department of Information Science and Engineering, NMAM Institute of Technology, Nitte (Deemed to be University), Nitte, India

**Keywords:** Sentiment analysis, Machine learning algorithms, Text classification, Convolutional neural network, K-fold cross validation, Health care, Engineering

## Abstract

This study examines end-user feedback on the Health and Wellness mobile health application, sourced from the play store, by integrating sentiment classification and linguistic intensity analysis to evaluate user perceptions. Comments are categorized into five distinct classes, ranging from highly positive (Class 5) to highly negative (Class 1), using keywords and linguistic patterns. The dataset comprises 20,651 rows, with 9063 highly positive comments, 5877 moderately positive comments, 1380 neutral comments, 298 moderately negative comments, and 4033 highly negative comments. To assess classification efficacy, several state-of-the-art algorithms were implemented, encompassing Support Vector Machines (SVM), Naive Bayes, ensemble-based classifiers (Decision Tree and Random Forest), and deep learning architectures (Convolutional neural networks, CNN). The experimental outcomes underscore the comparative advantage of these approaches in sentiment classification tasks, with CNN achieving the highest accuracy in detecting subtle contextual features. This analysis contributes to application design processes by providing insights into user interaction, satisfaction drivers and communication strategies for digital health platforms.

## Introduction

Sentiment analysis, formally characterized as computational opinion assessment, represents a specialized NLP methodology designed to systematically recognize, isolate and interpret emotional polarity or evaluative judgements manifested through unstructured linguistic data^1,2^. This method has become increasingly valuable for analysing app reviews, as it enables developers to gain actionable insights from user feedback, ultimately improving app performance and user satisfaction^3,4^. With the growing reliance on mobile applications in various domains, including healthcare, understanding user sentiments has become critical for enhancing app quality and addressing user concerns effectively.

The analysis of app reviews offers a wealth of information about user interaction modalities, preference distributions, and friction-inducing scenarios. By leveraging machine learning models, pre-processing techniques, and domain-specific, developers can extract meaningful insights from large datasets of user reviews. Advanced models such as Aspect-Based Sentiment Analysis(ABSA)^5^ and dependency-enhanced neural networks have shown promise in addressing the complexities in sentiment classification domains, notably for app reviews, these tools enable a deeper understanding of user sentiments, helping developers prioritize improvements and optimize app features.

In the realm of digital healthcare, mobile applications like HealthifyMe^6^ serve as critical enablers for advancing population health outcomes and holistic wellness. Analyzing user reviews of such apps provide valuable insights into user satisfaction, service quality, and areas for improvement. Online reviews serve as a rich source of feedback, offering perspectives from both healthcare customers (patients) and professionals. By examining these reviews, stakeholders can identify key determinants of satisfaction, such as usability, service quality, and payment processes, as well as pain points like in-app memberships or technical issues. This information is crucial for guiding platform enhancements, improving user trust, and optimizing healthcare outcomes.

To extract and analyse app review datasets, methodologies such as web scraping and text mining are commonly employed. Platforms like the Play Store^7,8^ provide a vast repository of user reviews, which can be systematically collected and analysed using advanced tools and techniques. Following data extraction, machine leaning classification techniques that incorporate grid search cross-validation can be used to tune hyperparameters, thus maximizing predictive classification efficacy for opinion mining^9^. These approaches ensure robust and reliable results, enabling developers and researchers to make data driven decisions.

This study focuses on conducting sentiment analysis of text reviews for the Health and Wellness app, a prominent digital healthcare platform. By employing advanced NLP techniques and machine learning models, we aim to uncover user sentiments, identify key factors influencing satisfaction, and provide actionable recommendations for app improvement. This study’s outcomes provide substantive contributions toward comprehending user experiences in digital healthcare services and offer insights for enhancing app performance and user engagement. This investigation systematically establishes the transformative potential of computational opinion mining in enhancing digital health interventions.

Despite the growing prevalence of mobile health applications, sentiment analysis in this domain faces several key challenges. First, there is a notable absence of publicly available, manually annotated datasets that capture the full spectrum of user sentiment, especially in a multi-class setting. Second, real-world app review data typically exhibits severe class imbalance, where extremely positive or negative sentiments dominate, limiting model generalization. Third, prior work in the field often focuses on binary classification or lacks systematic evaluation of multiple machine learning and deep learning models across rebalanced datasets. These gaps hinder the development of robust sentiment analysis systems tailored to health-related user feedback. This study addresses these challenges by introducing a manually annotated five-class sentiment dataset, applying augmentation and oversampling techniques to mitigate imbalance, and benchmarking both traditional and deep learning models to identify the most effective classification strategies.

## Literature survey

Polarity classification is commonly used in sentiment analysis to categorise text into various types of sentiments^2,10,11^. Sentiment analysis frequently employs several kinds of machine learning techniques, including Support Vector Machines (SVM), Logistic Regression, Decision Trees, and Naive Bayes^12–14^. To maximise the effectiveness of sentiment analysis models, efficient pre-processing techniques such as cleaning the data, normalising, and attribute engineering are essential^12^. To perform sentiment analysis on text reviews of an app, several key steps and methodologies can be employed, as evidenced by various studies in the field. Collect and pre-process user reviews by gathering feedback from app stores, then cleaning, normalizing, transforming, and integrating the data to enhance quality and relevance^15–17^.

Aspect Based Sentiment analysis models, such as BERT-based ones, extract sentiment triplets (aspect, opinion, polarity) from app reviews to provide detailed insights into user feedback, helping developers understand needs and enhance user experience^18^. SVM models, achieving high accuracy (e.g. 93.41 %percent with bi-gram + TF-IDF), and ensemble methods like Bagging can enhance classifiers such as Logistic Regression and Naive Bayes for app review classification^13,19^. The accuracy of sentiment analysis in app reviews can be increased by including domain-specific information, including software development terminology. To handle extremely fine sentiment assessment in this context, methods such as dependency-enhanced heterogeneous graph neural networks are being researched^20^ . Accurately capturing sentiment in app reviews requires understanding context and nuances, which can be challenging, while model performance varies according to data sets and applications, which requires continuous evaluation and fine-tuning to maintain accuracy^13,14^.

User reviews provide valuable insights into app performance, usability, and feature requests, often highlighting issues like crashes and responsiveness problems^21^. Reviews can be categorized to identify recurring issues and preferences, studies showing that high-rated apps have fewer crashes and responsiveness complaints compared to low-rated apps^22^. User reviews significantly impact app performance and developer actions, with studies showing that feedback influences downloads and faster responses enhance performance^23^. In addition, reviews that contain rich UX content are more likely to receive developer responses^24^. Convenience, performance, and security are key features in mobile payment apps that drive user satisfaction and market success^25–27^. Privacy and security concerns, such as data confidentiality and app permissions, are frequently highlighted in negative user reviews. Supervised and unsupervised machine learning methods were used to analyze user sentiment for payment apps. Most reviews were positive, but negative reviews tended to be longer and more detailed^28,29^. User experience factors such as ease of use, perceived value, and app design are critical in shaping user experiences and their subsequent reviews^27,30^. Sentiment analysis reveals that positive reviews highlight convenience and user-friendly interfaces, while negative reviews often cite security issues and poor customer service^25,26,31^. Performance expectancy, trust, and perceived security are key factors that shape users’ intentions to continue using mobile payment apps^32,33^.

Utilize web scraping tools such as Python-based libraries (e.g. Beautiful Soup, Regular Expression) to collect user reviews from the Play Store^34,35^. Data preprocessing involves cleaning (removing irrelevant data, handling missing values, normalizing text) and tokenization (splitting text into tokens)^36^. Feature extraction techniques include TF-IDF for textual features and collocation/dependency rules for identifying frequent and infrequent patterns^37,38^. Reviews are often unstructured, making extraction and analysis challenging^39^. Data collection involves extracting large datasets, such as 19,886 user reviews^40^ or over 429,000 reviews^34^ for popular apps. Cleaning involves removing irrelevant data and pre-processing text for quality, while feature engineering applies techniques like bag-of-words, TF-IDF, and chi-square to retain meaningful information^41^. Classification frameworks utilize tree-based algorithms and logistic regression to categorize product reviews on five-star or binary scales^42^.

Automatic classification methods leveraging semantic information and expert ratings, such as SCIBERT, have been developed to accurately evaluate the quality of scientific paper reviews^43^. Systematic literature reviews have identified various machine learning and rule-based approaches for classifying clinical reports, highlighting the importance of feature engineering and performance metrics^44^. Machine learning (ML) techniques are widely used for classification tasks across various domains. Classification in ML involves predicting a discrete label for input data, such as diagnosing diseases, categorizing images, or identifying spam emails^45,46^. Binary Classification predicts one of two classes (e.g. disease/no disease), while Multi-Class Classification predicts one of multiple categories (e.g. land cover types in satellite images)^47,48^. Feature selection enhances model performance by reducing overfitting and identifying relevant features using techniques like filter methods (statistical tests), wrapper methods (predictive models), and embedded methods (e.g. LASSO). Preprocessing steps, including normalization, data reduction, and handling missing values, are vital for preparing data for classification tasks^49,50^.

Quality and wide range of services are key factors in determining how satisfied patients are with telemedicine $(\beta = 0.5527)$, positive emotions are associated with service quality and offers, while negative emotions are associated with issues with payments $(\beta = -0.1173)$ as well as in-app subscriptions $(\beta = -0.031)$^51^. When choosing a consultant, patients give priority to category votes, online reviews (valence, volume), along with recommendation value^52^. The length and volume of reviews can impact patients’ decisions, moderated by the doctor-patient relationship^53^. Negative reviews, especially those with a high proportion and factual claims, significantly reduce patients’ selection intention^54^. The correctness of on-line physician reviews can be improved by reducing review variance through frequency of interaction, delivery of messages strategies, and clinical data.^55^. Social media interventions yield mixed outcomes in influencing health behaviors like physical activity, diet, and smoking cessation, with some studies showing significant improvements and others indicating minimal or no effect^56,57^. Through easily accessible health information and support, social media interventions can successfully reach at-risk groups, such as young people, the elderly, and people from low socioeconomic backgrounds, fostering health equity^58^. Nonprofits boost public engagement by combining functional interactivity, like providing information, with emotional strength, such as conveying powerful emotions^59^. Social media is used to build and maintain relationships with the community, foster community engagement, and support collective action^60^. The study developed an emotional lexicon for breast cancer patients, enhancing sentiment analysis and offering a tool to better understand and address their emotional states and needs for improved support^61^ . Grocery mobile app developers and managers can obtain important insights into client preferences as well as areas that require improvement by utilising text mining techniques, which will ultimately increase client fulfilment and loyalty^62–64^. Personalized recommendations and tailored shopping experiences based on user preferences can enhance satisfaction^65^. Sentiment analysis helps in understanding the emotional tone of reviews, distinguishing between positive and negative sentiments associated with different app features^66^. By examining customer-generated Google Play Store evaluations, this study investigates the adoption of mobile banking and the quality of its services, providing insights into important influencing factors^67^. Trust in the mobile banking system, perceived security, and user satisfaction are critical for adoption and serve as strong predictors of continued use and loyalty^68–73^. Enhancing service quality, ensuring security and usability, and delivering a positive user experience in mobile banking apps are key to boosting customer satisfaction and fostering loyalty^74–76^.

The performance of sentiment classification techniques has been examined using feature selection techniques as Unigram, Information Gain, Bigram, Chi-Square, as well as Gini Index^77^. Classification performance can be greatly enhanced by combining deep learning techniques like multi-layer perceptron deep neural networks with machine learning classifiers like logistic regression and random forests^78^. Classifying reviews into positive, negative, or neutral sentiments helps in understanding user satisfaction and areas needing improvement^79^. Grid Search optimizes hyperparameters for classifiers like SVM, Random Forest, and Naive Bayes to enhance accuracy and prevent overfitting^80^. With iteratively building models using k-1 samples and verifying on an additional subset, cross-validation—typically k-fold—is paired with Grid Search to robustly assess model performance and ensure generalisation to unseen data^81^. Naive Bayes and Logistic Regression classifiers benefit from Grid Search CV, demonstrating enhanced performance in tasks like emotion detection and sentiment classification^47,82^. Email spam has been successfully detected using natural language processing (NLP), and a variety of the preprocessing process, extraction of features, and machine learning techniques have helped create reliable spam filters^83^.

Transformer-based models, particularly BERT (Bidirectional Encoder Representations from Transformers), have shown significant promise in health-related sentiment analysis tasks. BERT can be fine-tuned for specific domains, such as healthcare, to improve its performance. For instance, models like BERT-Fuse, which combine BERT with other neural architectures, have shown high precision in mental health sentiment analysis^84,85^. A novel approach combines BERT for text analysis and Vision Transformer (ViT) for audio spectrograms to enhance depression diagnosis accuracy^86^. Transformer models, including BERT, have been utilized in predicting heart disease outcomes by analyzing sequential health data from wearable devices. This approach aids in early detection and prevention of heart-related events, potentially reducing hospital admissions^87^. Twitter-based sentiment analysis has been explored to gauge public health perception and patient satisfaction. Combining CNNs with LSTMs has shown superior performance in capturing the dynamic nature of Twitter data, achieving high accuracy, precision, recall, and F1 scores^88^. Twitter data is utilized to analyze consumer feedback on smartphones, providing insights into user satisfaction and product features. Transformer-based models like BERT have achieved high classification accuracy in this domain^89^. Deep learning models, including CNNs and LSTMs, are used to analyze patient sentiments on Twitter, offering a supplemental data stream to traditional survey-based approaches. These models can identify tweets related to patient experiences and sentiments, aiding in quality assessment and improvement^90^.

## Methodology

This study offers valuable insights by analysing app user sentiments at a detailed, granular level, uncovering specific preferences and challenges. These findings can empower app developers and managers to design more customer-centric services, enhance user satisfaction, and drive meaningful improvements in their applications. By focusing on nuanced feedback, businesses can better align their offerings with user expectations and foster long-term engagement. For performing sentiment analysis, we employed various machine learning algorithms, including Support Vector Machine(SVM), Naive Bayes, Random Forest and Decision Tree. Additionally, a deep learning model is develped based onConvolutional Neural Natworks (CNN) to enhance sentiment classification.

### Data collection

This study aims to analyse the sentiment of user reviews of the Health and wellness app. Python script was used to scrape online feedback from the Play Store of the mobile application, followed by data analysis. The dataset has 21,322 rows of text reviews that were specifically retrieved from the Play Store for the Health and Wellness app. The data collection period spans from April 30, 2021, to July 5, 2024. The extracted reviews include the following columns: review_id, content, score, thumbs_up, review_created_version, date, reply_content, and app_version. To maintain the quality and relevance of the data, multiple filtering conditions were employed. The extraction process is limited to English-language reviews and reviews submitted by Indian users, as clearly defined in the parameters of the extraction code. The use of the English language ensured consistency, enabling accurate analysis and interpretation of the data. The classification of the reviews into five sentiment categories was carried out manually, based on the presence of keywords related to sentiment and linguistic patterns, as described in Table 1. These keywords reflect varying degrees of emotional polarity and intensity, which guided the annotators in assigning class labels from 1 (very negative) to 5 (very positive). This process did not involve any automated or rule-based classification. Instead, human experts interpreted each review in context using predefined linguistic cues to ensure accurate labelling. The dataset scores were manually annotated^60,91^ by a human expert to ensure accurate labelling and to filter out irrelevant or low-quality reviews. To ensure annotation consistency and enhance data integrity, a second expert reviewed a randomly selected 40% of the dataset, chosen using a uniform distribution generator. The pseudocode for data extraction is given below:

- Step 1: Install the google-play-scraper package.
- Step 2: Import required libraries.
- Step 3: Define the app’s Play Store ID (app_id).
- Step 4: Fetch app details using app(app_id).
- Step 5: Extract all reviews using reviews_all() with specified parameters.
- Step 6: Convert reviews to a DataFrame.
- Step 7: Save the DataFrame to a CSV file.

A unified 5-point contentment assessment system is used by Google’s Play Store and is consistently applied to all apps on the app stores^62^. The least amount of satisfaction on this scale is represented by 1 star, and the maximum amount of satisfaction is represented by 5 stars^51^. This standardized system of rating makes it simple for consumers to provide feedback and helps developers accurately assess user sentiment. The maximum number of words in the review is 103. There are 2092 reviews with words between 1 and 5 and 297 reviews with words between 6 and 10. Remaining all reviews are above 10 words. The filtering conditions are given below: -

- Other languages text except English
- Unknown characters, words, symbols, names etc.
- Irrelevant words, meaningless words, sentence
- Words or sentences that are not related to the topic.

The reviews that satisfies any of these conditions are labelled 0 and manually removed from the extracted dataset. There were 670 reviews which is labelled as 0. The data set is manually annotated using the keywords available in the Table 1.

**Table 1:** Annotation conditions: Table contains keywords which determine the various class labels, its description, review examples and its total count.

Keywords	Class label	Description	Example	Count
Words with strong adjectives: *Egs:- very, amazing, great, top, essential, awesome, trust, excel*	5	Positive comments that use strong adjectives to emphasize satisfaction or admiration.	“This app is *amazing*! It has completely transformed my daily routine.” “The course is really *great* and easy to use.”	9063
Positive comments without strong adjectives	4	Express positive comments that show satisfaction or agreement but do not use strong adjectives.	“The app is helpful for tracking meals and workouts.” “How I like the reminders; they keep me on track!”	5877
Neutral comments	3	Comments that are neither strongly positive nor strongly negative. They may include neutral statements or suggestions.	“I do see some improvements.” “The app is okay, but it could use better instructions.”	1380
Negative comments without strong adjectives	2	Comments that express dissatisfaction or disagreement but do not use strong adjectives.	“The app crashes often, and it’s frustrating.” “The categorization is incomplete and inaccurate.”	298
Negative comments with strong adjectives (*completely useless, etc.*)	1	Highly negative comments that use strong adjectives to emphasize extreme dissatisfaction or anger.	“This app is *completely useless*; I don’t even know why I downloaded it!” “I’m *really disappointed* with the purchase. It’s not worth the money.”	4033

Workflow for Preparing a standard dataset with ground truth

**Fig. 1:**
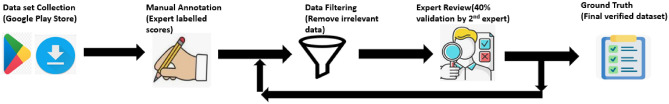
Workflow for preparing a standard dataset.

The process of constructing a high-quality dataset with reliable ground truth involved the following systematic steps and its diagrammatic representation is given in Fig. 1

1. Dataset collection – Data was gathered from the Play Store, ensuring a diverse representation of user reviews.
2. Manual annotation – A human expert carefully assigned scores to the reviews to ensure accurate labelling. enumerate environment.
3. Data filtering – The dataset was refined by removing irrelevant and low-quality data to enhance integrity.
4. Expert review – To maintain annotation reliability, 40% of the total dataset was independently reviewed by a second expert.
5. Ground truth establishment – The final dataset, incorporating expert validation, was standardized as ground truth for further analysis and model training.

### Dataset description and model building

The above Table: -1 presents the class-wise distribution of manually annotated reviews: 4033 (Class-1), 298 (Class-2), 1380 (Class-3), 5877 (Class-4), and 9063 (Class-5). The dataset contains two columns: content (text data) and label (class labels ranging from 0 to 5). Naïve Bayes, Support Vector Machine (SVM), Decision Tree and Random Forest are among the text categorisation algorithms used in this study’s supervised machine learning pipeline. The dataset, stored in an excel file, undergoes a pre-processing phase to ensure data integrity by removing entries with missing values in the text(content) or label columns. Text pre-processing included lowercasing, removal of punctuation and special characters, stop-word removal using the NLTK stop-word list, and lemmatization using the spaCy library. This ensured that the input to the TF-IDF vectorizer and CNN tokenizer was semantically consistent and free of irrelevant tokens. The content column serves as the feature set, while the label column represents class labels. To facilitate model training and evaluation, the dataset is partitioned into training (70%) and testing (30%) subsets using stratified sampling, ensuring proportional class distribution across both sets.

With a predetermined vocabulary size and output restricted to the top 5000 most important terms, the Term Frequency- Inverse Document Frequency (TF-IDF) vectorizer is used to transform textual input into a numerical form appropriate for machine learning models^7^. The TF-IDF method lessens the influence of high frequency, less informative phrases by allocating weights to terms according to their relevance in the collected data. Compared to traditional Bag-Of-words models, TF-IDF provides a more effective feature representation by emphasizing informative words and mitigating redundancy. This transformation is essential for training models on textual data and enhancing classification performance.

Following feature extraction, multiple machine learning models are trained and evaluated on the TF-IDF transformed dataset. The SVM classifier is employed due to its effectiveness in handling high dimensional feature spaces and its ability to find an optimal decision boundary for text classification. Furthermore, Decision Tree and Random Forest classifiers are incorporated to analyse model interpretability and the benefits of ensemble learning, while naïve Bayes serves as a probabilistic baseline model. Standard assessment metrics, including as accuracy, F1-score, recall, and precision, are calculated to evaluate each classifier’s predicted ability. Additionally, confusion matrix visualization is utilized to analyse class-wise performance and misclassification patterns. The entire classification pipeline is implemented in Python using Sci-kit Learn library, and the results are analysed to compare the efficacy of different classifiers in text classification tasks.

The original data set was evaluated using several machine learning techniques (Table 2). However, due to severe class imbalance, especially in Classes 2 and 3, random oversampling was employed. This involved replicating minority class samples to ensure all classes had equal representation (9,063 samples), resulting in a balanced dataset used for further analysis (Table 3). In addition, a series of five targeted data augmentation stages were implemented to systematically enrich the minority classes and examine their impact on classification performance. Augmentation was conducted via synonym replacement, where sentiment-preserving alternatives were substituted for select keywords in reviews, thereby increasing diversity while maintaining contextual integrity. The augmentation steps were designed as follows:

1. *Augmented 1:* Class 2 and Class 3 were doubled using synonym replacement.
2. *Augmented 2:* Additional 1500 synthetic reviews were added to both Class 2 and Class 3.
3. *Augmented 3:* Class 3 was further enriched with 1000 samples.
4. *Augmented 4:* Class 2 was enriched with 1000 additional samples.
5. *Augmented 5:* Both Class 2 and Class 3 were augmented with 2500 synthetic samples each.

These datasets were subsequently used to train and evaluate all classification models. The objective of this staged augmentation was to assess whether incremental enrichment of underrepresented classes improves model performance and to identify the point of diminishing returns (Refer Table 4 to Table 8).

### Proposed convolutional neural network (CNN) architecture

**Fig. 2:**
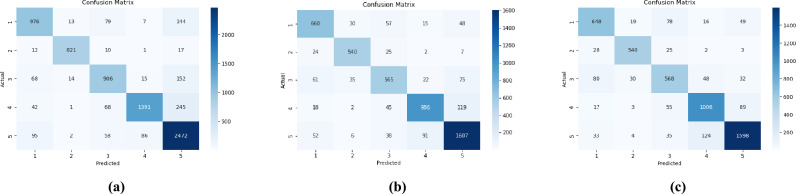
(**a**) Confusion matrix of Augmented 5 data set using RF(best performing model), (**b**) Confusion matrix of CNN model using Augmented 5 dataset, (**c**) Confusion matrix of refined CNN model using Augmented5 dataset.

**Fig. 3:**
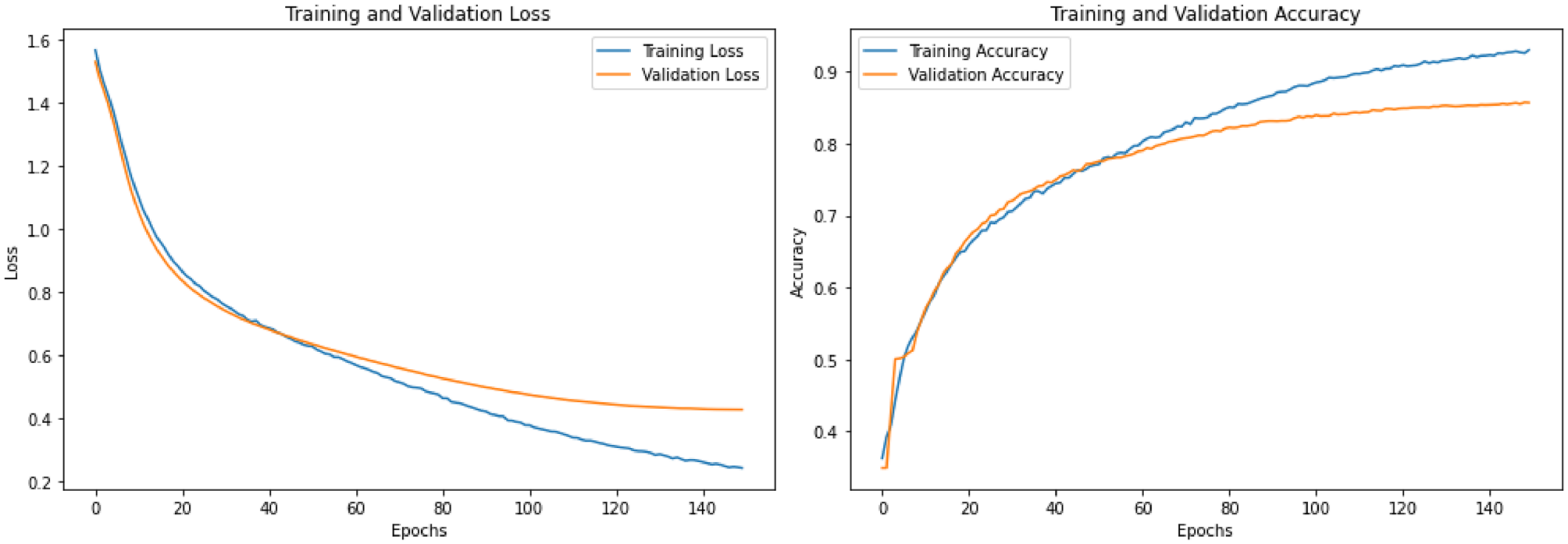
Loss and accuracy plot of CNN.

**Fig. 4:**
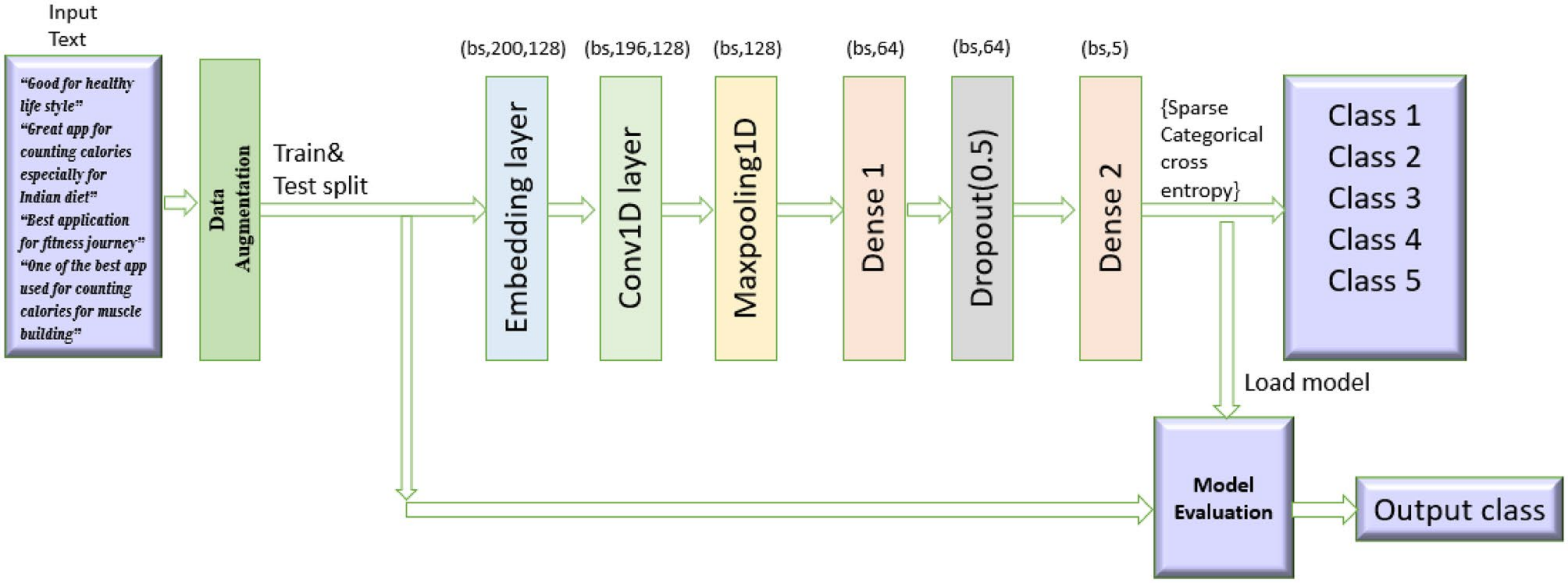
Proposed CNN architecture(batch size(bs)=32).

The code uses the Keras Sequential API to create a Convolutional Neural Network (CNN) architecture intended for text classification. The workflow starts with tokenization and padding, in which the Keras Tokenizer is used to transform the text data into numeric sequences up to a 5000-word vocabulary limit(max_words=5000). To guarantee consistent input dimensions to the model, these sequences are subsequently padded with a constant length of 200 (max_len=200).The model begins with an Embedding layer that captures semantic links between words by mapping integer-encoded word sequences to dense 128-dimensional vector. A 1D Convolutional Layer (Conv1D) of 128 filters with a kernel depth of 5 follows thereafter, employing the ReLU activation function to extract local patterns (such as n-grams) from the embedded sequences. A Global Max Pooling Layer is then applied to reduce the spatial dimensions by retaining the most significant features from each filter, effectively summarizing the extracted patterns. For learning the highest-level representations, the pooled features are run through a dense Layer having 64 neurons utilising ReLU activation. To reduce overfitting, a Dropout Layer at a rate around 0.5 is the applied. Lastly, this Output Layer generates class probabilities for the five target classes using softmax activation function with five neurons. The model is compiled using the Adam optimizer with a low learning rate (0.00001) and trained using sparse categorical cross-entropy loss, ensuring efficient learning and robust performance for multiclass text classification. This architecture is computationally efficient, leverages local text patterns effectively, and incorporates regularization techniques to enhance generalization. The CNN model achieved an accuracy of 84.95%, with an F1 score of 84.91%, recall of 84.95%, and precision of 84.92%, demonstrating strong and consistent performance across all metrics. Fig. 2b shows the confusion matrix for CNN model. The loss and accuracy plots in Fig. 3 exhibit stable convergence, with training and validation curves closely aligned, indicating effective learning and generalization without overfitting. This demonstrates the model’s robustness, achieved through Dropout regularization, low learning rate optimization, and a well-designed CNN architecture. The CNN architecture is shown in the Fig. 4.

The CNN architecture is refined to introduce several key enhancements compared to the previous version. First, it incorporates two 1D Convolutional Layers (each with 64 filters and L1L2 regularization) instead of one, allowing for deeper feature extraction from the text. Layer Normalization is added after the first convolutional layer, replacing Batch Normalization, to stabilize training and improve convergence. The model also includes additional Dropout Layers with adjusted rates (0.4 after the first convolutional layer and 0.3 after the dense layer) to further mitigate overfitting. A second Dense Layer with 32 neurons is introduced to learn more complex representations before the output layer. Additionally, the model uses L1L2 regularization in all convolutional and dense layers to penalize large weights and improve generalization. Finally, call-backs like Early stopping and ReduceLROnPlateau are integrated to optimize training by halting early ,if performance plateaus and dynamically reducing the learning rate, respectively. These changes collectively enhance the model’s robustness,depth, and ability to generalize.

**Fig. 5:**
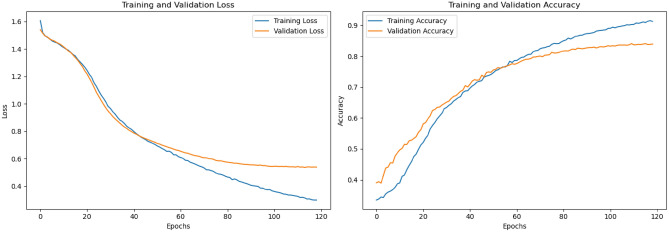
Loss and accuracy plot of refined CNN model.

The refined model also achieves high performance metrics, with an accuracy of 84.99%, F1 score of 85.00%, recall of 84.99%, and precision of 85.02%, demonstrating its effectiveness for the classification task. The confusion matrix is shown in Fig. 2c. The loss and accuracy plots Fig. 5 demonstrate excellent convergence and stability, with training and validation curves closely aligned, indicating effective learning and generalization. This is achieved through strong regularization (Dropout, L1L2), Layer Normalization, and callbacks (Early Stopping, ReduceLROnPlateau), ensuring optimal performance without overfitting.

## Results and discussion

**Fig. 6:**
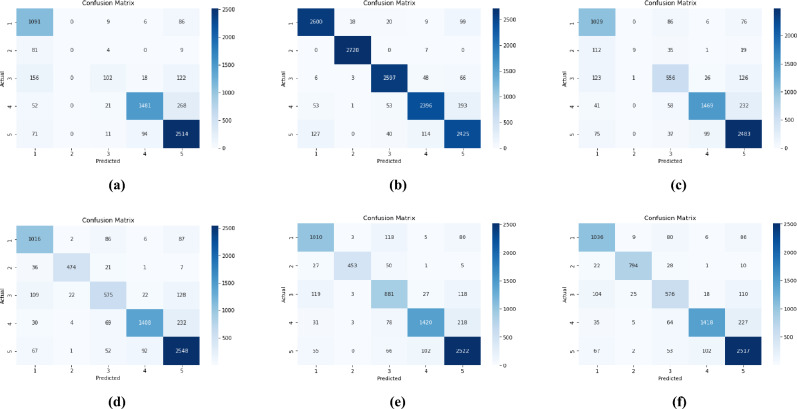
Confusion matrix of best performing models with data augmentation: (**a**) original data using SVM, (**b**) oversampled data set using RF, (**c**) Augmented1 data set using SVM, (**d**) Augmented2 data set using SVM, (**e**) Augmented3 data set using SVM, (**f**) Augmented4 data set using SVM.

The performance metrics of various machine learning classifiers using original dataset are presented in Table 2. The classification models, including Naïve Bayes, Decision tree, SVM, and Random Forest, achieved accuracies of 81.18%, 77.93%, 83.73%, and 82.21% respectively. Among these models, SVM demonstrated the best performance, achieving the highest accuracy of 83.73 and an F1-score of 82.12%. This superior performance highlights the effectiveness of SVM in handling the classification task with improved predictive capability. Confusion matrix of original data set using SVM (best performing model)is given in Fig. 6a

**Table 2:** Performance comparison for original dataset.

Metrics (%)	Classifiers			
	Naïve Bayes	Decision Tree	SVM	Random Forest
Accuracy	81.18	77.93	83.73	82.21
Precision	81.85	77.07	82.55	81.37
Recall	81.18	77.93	83.73	82.21
F1-score	81.90	77.45	82.12	80.60

The performance metrics of various machine learning classifiers evaluated on the oversampled dataset are presented in Table 3. The classification models, including Naïve Bayes, Decision Tree, SVM, and Random Forest, achieved accuracies of 81.56%, 91.71%, 93.61%, and 93.70%, respectively. Among these models, Random Forest exhibited the best performance, achieving the highest accuracy of 93.70% along with an F1-score of 93.70%, demonstrating its effectiveness in handling the classification task. Confusion matrix of oversampled data set using SVM (best performing model) is given in Fig. 6b

**Table 3:** Performance comparison for oversampled dataset.

Metrics (%)	Classifiers			
	Naïve Bayes	Decision Tree	SVM	Random Forest
Accuracy	81.56	91.71	93.61	93.70
Precision	81.63	91.66	93.61	93.72
Recall	81.56	91.71	93.61	93.70
F1-score	81.46	91.66	93.61	93.70

The performance metrics of various machine learning classifiers evaluated on the “Augmented1” dataset are presented in Table 4. The classification models, including Naïve Bayes, Decision Tree, SVM, and Random Forest, achieved accuracies of 77.01%, 76.16%, 82.79%, and 81.95%, respectively. Among these models, SVM demonstrated the best performance, attaining the highest accuracy of 82.79%, along with an F1-score of 81.82%, highlighting its effectiveness in handling the classification task. The confusion matrix of Augmented1 data set using SVM (best performing model)is given in Fig. 6c

**Table 4:** Performance comparison for Augmented 1 dataset.

Metrics	Classifiers			
	Naïve Bayes	Decision Tree	SVM	Random Forest
Accuracy	77.01	76.16	82.79	81.95
Precision	77.11	75.70	83.30	82.74
Recall	77.01	76.16	82.79	81.95
F1-score	74.57	75.87	81.82	81.63

The performance metrics of various machine learning classifiers evaluated in the “Augmented2” data set are summarized in Table 5. The classification models, including Naïve Bayes, Decision Tree, SVM, and Random Forest, achieved accuracies of 75.56%, 76.80%, 84.86%, and 84.86%, respectively. Among these models, SVM exhibited the best performance, attaining the highest accuracy of 84.86%, along with an F1 score of 84. 79%, demonstrating its effectiveness in the classification task. The confusion matrix of Augmented2 data set using SVM(best performing model) is given in Fig. 6d

**Table 5:** Performace comparison for Augmented 2 dataset.

Metrics	Classifier			
	Naïve Bayes	Decision Tree	SVM	Random Forest
Accuracy	75.56	76.80	84.86	84.86
Precision	78.05	76.46	85.06	84.37
Recall	75.56	76.80	84.86	84.03
F1-score	73.57	76.54	84.79	83.88

The performance metrics of various machine learning classifiers assessed on the “Augmented3” dataset are summarized in Table 6. The classification models, including Naïve Bayes, Decision Tree, SVM, and Random Forest, achieved accuracies of 75.47%, 77.17%, 85.00%, and 84.67%, respectively. Among these models, SVM demonstrated the best performance, achieving the highest accuracy of 85.00%, along with an F1-score of 85.02%, highlighting its effectiveness in the classification task. The confusion matrix of Augmented3 data set using SVM (best performing model) is given in Fig. 6e

**Table 6:** Performance comparison for Augmented 3 dataset.

Metrics	Classifier			
	Naïve Bayes	Decision tree	SVM	Random Forest
Accuracy	75.47	77.17	85.00	84.67
Precision	77.97	76.91	85.00	85.14
Recall	75.47	77.17	85.36	84.67
F1-score	73.65	76.96	85.02	84.63

The performance metrics of machine learning classifiers on the “Augmented4” dataset (Table 7) show that SVM outperformed other models, achieving the highest accuracy of 85.74% and an F1-score of 85.69%, demonstrating its effectiveness in classification. The confusion matrix of Augmented3 data set using SVM (best performing model) is given in Fig. 6f

**Table 7:** Performace comparison for Augmented 4 dataset.

Metrics	Classifier			
	Naïve Bayes	Decision Tree	SVM	Random Forest
Accuracy	77.77	77.63	85.74	84.81
Precision	79.48	77.38	85.92	85.31
Recall	77.77	77.63	85.75	84.81
F1-score	76.51	77.42	85.69	84.67

**Fig. 7:**
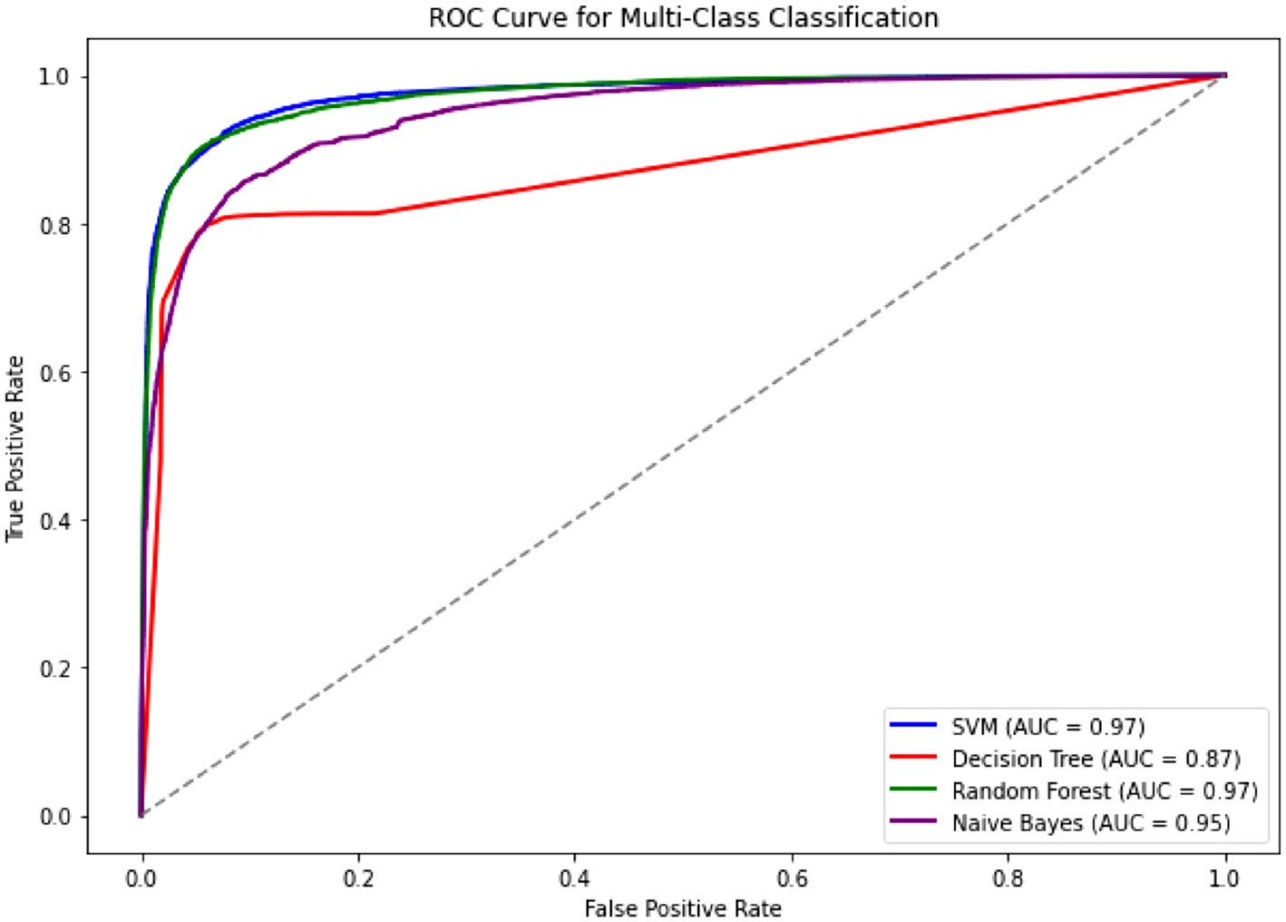
ROC curve for multiclass classification (Augmented 5 dataset).

The performance metrics of machine learning classifiers on the “Augmented5” dataset (Table 8) indicate that Random Forest achieved the highest accuracy of 85.33% and an F1-score of 85.30%, demonstrating its superior effectiveness in the classification task. The confusion matrix of Augmented5 data set using RandomForest(best performing model) is given in Fig. 2a. The ROC curve plotted for Augmented5 dataset is presented in Fig. 7. The AUC values indicates that SVM and random forest achieved the highest performance both with an AUC 0.97.

**Table 8:** Performance comparison for Augmented 5 dataset.

Metrics	Classifier			
	Naïve Bayes	Decision Tree	SVM	Random Forest
Accuracy	77.65	77.49	84.85	85.33
Precision	79.34	77.49	86.26	85.70
Recall	77.65	77.29	85.85	85.33
F1-score	77.10	77.34	85.91	85.30

**K-fold cross validation results**

To evaluate the generalization performance of various machine learning models, 3-fold, 5-fold, and 7-fold cross-validation were applied, and the corresponding mean accuracy is presented in Table 9. K-fold cross validation partitions the dataset into K equally sized subsets, where the model is trained on K-1 folds and tested on the remaining fold in an iterative manner. This process is repeated k times, ensuring that each fold serves as a test set once. The application of 3,5,7 folds allows for a comprehensive evaluation by mitigating the risks of overfitting and underfitting, while the mean accuracy of all folds provides a robust consistent estimate of model performance. The results show that SVM and Random Forest are the most effective models, with SVM slightly outperforming in accuracy. The performance improvement with more folds highlights the need for sufficient training data to enhance generalization, offering valuable insights for model selection in similar tasks.

**Table 9:** K fold cross validation results.

K-fold cross validation	Mean accuracy (percentage)			
	SVM	Decision Tree	Random Forest	Naïve Bayes
3-Fold CV	86.00	77.29	85.48	78.35
5-Fold CV	86.56	78.49	86.17	79.37
7-Fold CV	86.85	79.02	86.65	79.82

Random Forest was optimized using grid search with hyperparameters including n_estimators {50, 100, 200}, max_depth {None, 10, 20, 30}, min_samples {2, 5, 10}, min_samples_leaf {1, 2, 4}, and bootstrap {True, False}, employing 5-fold cross-validation. Among these combinations, the model achieved its best performance with the following optimal parameters when applied to different types of augmented datasets is shown in Table 10. The Random Forest model, optimized via grid search, achieved its best cross-validation accuracy of 84.94% on Augmented5, with optimal parameters including bootstrap=False, max_depth=None, min_samples_leaf=1, min_samples_split=5, and n_estimators=100, highlighting the impact of hyperparameter tuning and data augmentation on model performance.

**Table 10:** Random forest grid search CV best parameters.

Data set	Best parameters					
	bootstrap	max_ depth	min_samples _leaf	min_samples_ split	n_estimators	Best cross-validation accuracy
Original	False	None	1	2	200	81.74%
Oversampled	False	None	1	2	200	98.31%
Augmented 1	False	None	1	5	100	82.70%
Augmented 2	False	None	1	5	200	83.94%
Augmented 3	False	None	1	2	200	84.57%
Augmented 4	False	None	1	5	100	84.31%
Augmented 5	False	None	1	5	100	84.94%

Table 11 provides a consolidated overview of the top performing classification models across different data preparation strategies, including the original, oversampled, and augmented datasets, as well as a CNN deep learning approach. The random forest classifier, when applied to the oversampled dataset, achieved the highest overall performance, with an accuracy and F1-score of 93.70%, demonstrating its superior capability in addressing class imbalance and enhancing predictive accuracy. Among the augmented datasets, the SVM model trained on Augmented 4 exhibited the best performance, attaining an accuracy of 85.74% and an F1-score of 85.69%, indicating the efficacy of synthetic data augmentation in improving model generalizability. The CNN model, trained on the Augmented 5 dataset, also delivered robust results, achieving an accuracy of 84.95%, F1 score of 84.91%, and balanced precision and recall values, reflecting its effectiveness in learning complex patterns from augmented input representations. These comparative results provide a clear indication that while oversampling with random forest yielded the highest scores, both data augmentation and CNN based modeling also achieved competitive performance.

Although the augmented datasets were constructed sequentially from Augmented1 to Augmented5, the intention was not to induce a guaranteed progressive improvement in classification performance. Instead, each augmentation step was designed to experimentally assess the impact of selectively increasing samples in underrepresented classes, particularly class 2 and class 3. The best performance among the augmented datasets was observed in Augmented4, where only class 2 was augmented, suggesting that a more targeted augmentation approach can be more effective than broadly increasing synthetic data. In contrast, Augmented5, which added 2500 synthetic samples to both class 2 and class 3, showed a slight decline in accuracy. This highlights the phenomenon of diminishing returns, where excessive augmentation may lead to redundancy, reduced data variability, or overfitting to synthetic patterns. These results suggest that strategic augmentation of minority classes, rather than uniform or excessive expansion, is more beneficial for achieving optimal classification outcomes.

**Table 11:** Comparative summary of best performing models.

Dataset type	Best performing model	Accuracy(%)	F1-score (%)	Precision (%)	Recall (%)
Original	SVM	83.73	82.12	82.55	83.73
Oversampled	Random Forest	93.70	93.70	93.72	93.70
Augmented 1	SVM	82.79	81.82	83.30	82.79
Augmented 2	SVM	84.86	84.79	85.06	84.86
Augmented 3	SVM	85.00	85.02	85.00	85.36
Augmented 4	SVM	85.74	85.69	85.92	85.75
Augmented 5	Random Forest	85.33	85.30	85.70	85.33
Augmented 5	CNN	84.95	84.91	84.92	84.95

## Conclusion

This study offers an in-depth assessment of customer feedback for the Health and Wellness app, using innovative deep learning and machine learning methods to accurately categorise sentiments. Using models like Convolutional Neural Networks (CNN), Random Forests, Decision trees, Support Vector Machines (SVM), and Naïve Bayes, the study shows how well various algorithms handle text-based assessment of sentiment. With SVM attaining the greatest accuracy of 85.74% on the augmented datasets and CNN reaching 84.99% accuracy with optimised hyperparameters, the results show that SVM and CNN are best performing models. This study makes several key contributions to the domain of mobile health sentiment analysis. First, it introduces a manually annotated, domain specific dataset that enables fine-grained, multi-class classification of user sentiment in the Health and Wellness domain. Second, a comparative evaluation of both traditional machine learning algorithms(Naïve bayes, Decision tree, random forest, and SVM) and a deep learning architecture (CNN) was performed to assess their effectiveness across different data configurations. Third, the study systematically applies oversampling and controlled data augmentation strategies to address class imbalance, improving model generalization and performance stability. By using data augmentation and oversampling, the study also tackles class imbalance, greatly enhancing model performance and generalization.

The research objective, which was to develop an accurate and robust sentiment classification pipeline for Health and Wellness app reviews, was accomplished through a systematic methodology. This included comprehensive data pre-processing, manual labelling, rebalancing via augmentation and oversampling, and extensive model evaluation. App developers and managers can more effectively understand customer preferences and issues by utilizing the outcomes, which offer practical insights. This study provides a means of improving user experience while promoting sustained engagement by emphasising important areas of customer satisfaction as well as discontent. This study makes an important advancement to the discipline of sentiment assessment because of the methodological approach taken to collecting data, the pre-processing phase and model validation, which guarantees the precision and repeatability of the findings.

### Future work

While the current work focused on CNN and ensemble models, future research could explore transformer-based architectures such as BERT or Ro BERT a for improved contextual understanding. Further extensions could include multi-lingual sentiment analysis and aspect-based classification to better capture domain-specific feedback for health applications.

Future studies will seek to enhance the interpretability of sentiment classification models by integrating Explainable AI (XAI) techniques such as Local Interpretable Model-Agnostic (LIME) and Shapley Additive exPlanations (SHAP). The tools can provide insight into model predictions by identifying the most influential features for each decision, thereby improving the transparency and trustworthiness of sentiment analysis in healthcare-related applications.

## Data Availability

The data supporting the findings of this study are available from the corresponding author upon reasonable request.
